# Intraoperative margin assessment by wireless signals in thoracoscopic anterior (S3) segmentectomy using a radiofrequency identification marker

**DOI:** 10.1007/s11748-021-01762-7

**Published:** 2022-01-22

**Authors:** Yojiro Yutaka, Akihiro Ohsumi, Daisuke Nakajima, Masatsugu Hamaji, Toshi Menju, Hiroshi Date

**Affiliations:** Department of Thoracic Surgery, Kyoto University Hospital, 54 Kawaharacho, Shogoin, Sakyo-ku, Kyoto, 606-8507 Japan

**Keywords:** Localization, Marking, Segmentectomy, Surgical margin, Thoracoscopy

## Abstract

Despite the use of near-infrared thoracoscopy with intravenous indocyanine green, intraoperative assessment of the surgical margin for the resection of non-palpable tumors located near the intersegmental plane requires highly advanced surgical skill for the prevention of local recurrence. Because the demarcation line is limited to the pleural surface, to overcome uncertainty in tumor palpation for deeply located small-sized lesions, other supplemental localization techniques have been proposed. Here, we present a novel surgical technique using radiofrequency identification markers for intraoperative assessment of the lateral surgical margin in segmentectomy.

**Supplementary Information:**

The online version contains supplementary material available at 10.1007/s11748-021-01762-7.

## Introduction

Anatomical segmentectomy can achieve oncologic outcomes similar to lobectomy in small-sized lung cancer [1]. Recent advances in imaging technology have allowed precise preoperative simulation using 3D imaging and facilitated segmentecomy [2]. If insufficient surgical margins are expected on the basis of 3D simulation, the surgical plan should be changed from single segmentectomy to additional resection with adjacent subsegmentectomy [3]. However, complicated segmentectomy requires advanced surgical skills and can hinder the matching of intraoperative anatomy with the simulated image; therefore, simple segmentectomy with additional resection of the adjacent segment beyond the affected area may be alternatively selected. In such cases, instead of simplification of the surgical procedure in segmentectomy, the resection line should be adjusted to the tumor location [4]. We recently reported a novel surgical technique using radiofrequency identification (RFID) markers in wedge resection for determination of deep surgical margins [5, 6]. This localization technique using wireless signals can also be applied for segmentectomy for determination of lateral resection margins. In this paper, we describe the technical aspects of this procedure.

## Technical description

The case presented here is a 79-year-old female with a history of angina pectoris. A sub-centimeter pure ground-glass opacity progressed to a part-solid lesion within 6 years. Although segmentectomy was suitable, the tumor location, which was near the intersegmental plane between S3a and S2b, would have caused problems for securing the surgical margin for typical S3 segmentectomy.

### Preoperative assessment of the surgical margin by 3D-computed tomography (CT)

**Fig. 1:**
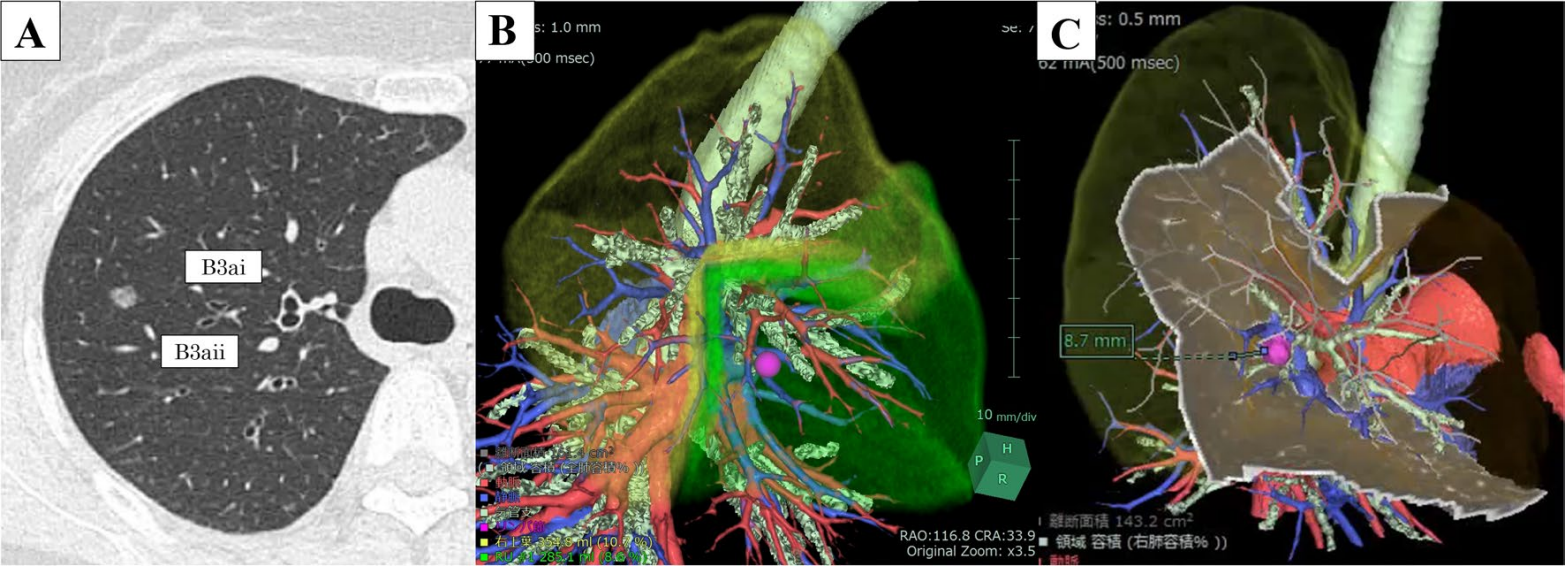
Preoperative computed tomography (CT) and simulation of the surgical margin using a three-dimensional (3D) image. **A** The lesion is located at S3a. **B** The pink mark indicates the tumor position, and the green area represents the target segment. **C** An 8.7-mm surgical margin was ensured by single S3 segmentectomy

A lesion featuring a part-solid nodule (7 mm) was located at the lateral sub-segment of the anterior segment (S3a) (Fig. 1A). Preoperative 3D imaging revealed that the lesion was located near the segmental plane, between S2b and S3a, and the simulated surgical margin was only 8.7 mm by single S3 segmentectomy (Fujifilm Medical, Tokyo, Japan) (Fig. 1B, C). Therefore, we performed S3 segmentectomy with additional resection of S2b beyond the intersegmental plane using an RFID marker.

### Cone-beam CT (CBCT)-guided RFID marking procedure

**Fig. 2:**
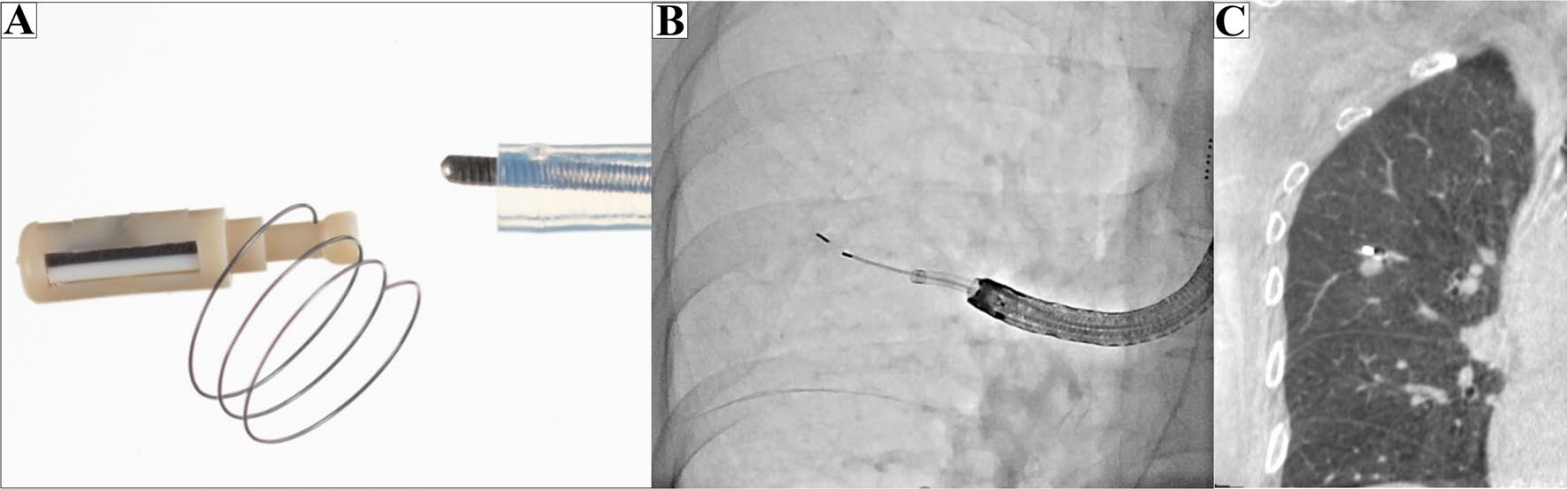
Radiofrequency identification (RFID) marking procedure using cone-beam computed tomography (CBCT) in a hybrid operation theater. **A** Passive RFID tag (3.2 × 1.6 × 0.9 mm). **B** Intraoperative fluoroscopy. **C** Preoperative CT showing an RFID marker placed 3 mm from the lesion

The RFID system (SuReFInD; Hogy Medical Co., Ltd., Tokyo, Japan) consists of the following components: (1) a delivery device for RFID markers with 5-mm NiTi coil anchors (Fig. 2A); (2) a wand-shaped probe (10-mm diameter, 30-mm effective range). The detailed algorithms used in the system were previously reported [7, 8]. Briefly, RFID markers (passive transponders with no built-in battery) are activated by the electromagnetic field produced by a probe acting as both the power supply and receiver antenna. The strength of the signal received by the probe is converted to five gradual changes in sound pitch by the signal processing unit, the pitch becoming higher as the probe approaches the marker. The marker is located using a detection probe via wireless communication followed by tone changes in accordance with the marker–probe distance.

Marker placement was performed in a hybrid operation theater using CBCT prior to surgery (Artis Zeego; Siemens Healthcare, Erlangen, Germany). Under general anesthesia, a BF-1T260 bronchoscope (Olympus, Tokyo, Japan) was inserted via the intubation tube and an RFID delivery catheter was advanced close to the target. Following two repetitions of CT using electromagnetic navigation bronchoscopy (ENB) (SuperDimension™ navigation system, Version 7.0; Medtronic, Minneapolis, MN, USA), the RFID marker was released under fluoroscopic view (Fig. 2B). In the current case, preoperative CT confirmed that the marker was located 3 mm from the lesion without any complications (Fig. 2C). The bronchoscopy procedure time was 14 min.

### Intraoperative assessment of the surgical margin by wireless signals

**Fig. 3:**
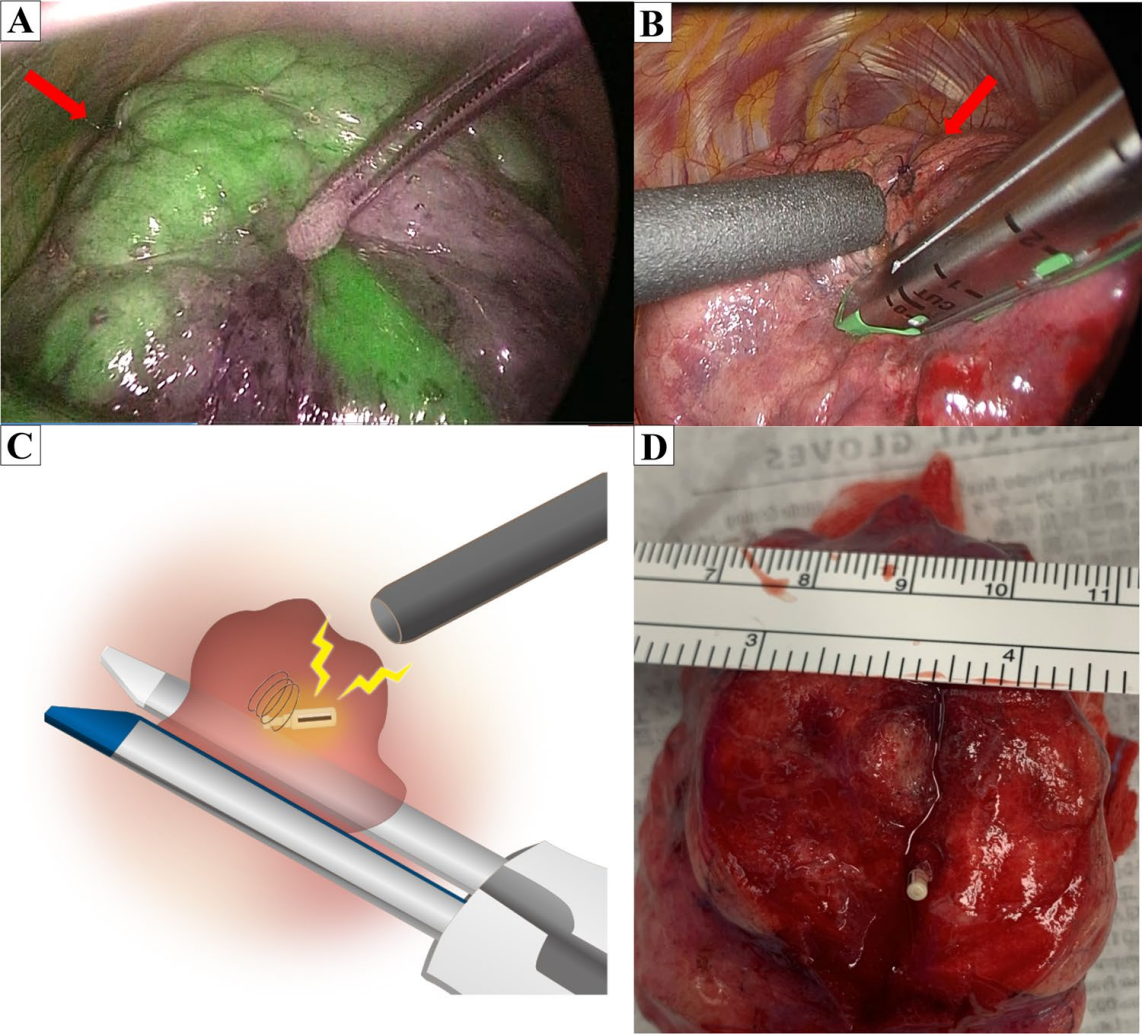
Intraoperative near-infrared image after intravenous indocyanine green injection. **A** The demarcation revealed the tumor located outside the undyed area. The red arrow indicates the marker position, which is located 3 mm from the tumor between B3ai and B3aii. **B** The resection line was adjusted according to the tumor location indicated by wireless communication. **C** The location of the marker can be easily confirmed in the targeted segment by wireless communication. **D** Macroscopic findings. This lesion was finally diagnosed as a 7-mm papillary adenocarcinoma, and it was removed with a margin of 12 mm

Tumors were recovered without palpation of the lung. Segmentectomy was performed via three ports: fourth intercostal space at the anterior axillary line (40 mm), seventh intercostal space at the mid-axillary line (15 mm), and in the triangle of auscultation (15 mm). The lobe of interest was scanned with the locating probe from the anterior port. Because the probe has directivity in wireless communication, the angle of incidence between the probe and the pleural surface approached 90° during marker exploration by the probe. This detection revealed the nearest pleural point from the marker. Operators located the marker within 15 s by following tone changes in accordance with the marker–probe distance. Following localization, a 4-0 polydioxanone stitch was placed at a pleural surface point near the marker. According to the preoperative simulation, prior to A3 division, a sentinel lymph node was intraoperatively confirmed as negative by frozen section analysis. Following division of V3 and B3 to evaluate the intersegmental plane, the operative lung was fully inflated by the anesthesiologist to the subsequent demarcation of the intersegmental plane to improve the intrapulmonary blood flow in the deflated lung, in addition to an intravenous injection of 0.25 mg/kg indocyanine green. This revealed that the 4-0 stitch was outside the undyed area to be resected; therefore, tumor position was relocated by RFID marker detection in the inflated lung to assess surgical margins (Fig. 3A). The resection line was adjusted during linear stapler clamping toward S2b in accordance with the tumor location (Fig. 3B, C). Despite the deep tumor position, the location of the marker was easily confirmed during lung division, and wireless signals from the marker were confirmed in the targeted segment, which resulted in a secured margin of 12 mm (Fig. 3D; Supplemental Video). The postoperative course was uneventful, and the chest drain was removed on postoperative day 3.

## Discussion

To overcome uncertainty in tumor palpation, preoperative simulation using 3D-CT has been utilized for the measurement of margins between the tumor and planned resection line. After appropriate resection of the targeted hilar structures, near-infrared fluorescence imaging can achieve 84.0–95.6% clear demarcation of the intersegmental line according to intrapulmonary blood flow [9]. However, the detection of demarcation lines can become difficult in some complicated segmentectomies, as well as in some pulmonary conditions, including emphysema and anthracosis. In the current case, the presence of multiple collateral vessels, which could not be preoperatively identified on 3D-CT, may have caused a reduction in the undyed area in comparison to the virtual segmental area simulated from the preoperative pulmonary artery structure.

This novel wireless localization technique requires bronchoscopic placement of RFID markers near the tumor. Although intrapulmonary sites accessible via bronchoscopy depend on an individual’s bronchial anatomy, this critical issue can be overcome using CBCT-guided bronchoscopy. In the current case, the RFID marker was placed under CBCT-guided bronchoscopy in combination with ENB because the tumor had no apparent bronchus sign [10]. Regarding the capability of bronchoscopy to reach peripheral pulmonary lesions, newer technologies have been introduced to improve the rate of successful bronchoscopic navigation to peripheral pulmonary nodules [11]. ENB was clinically approved for preoperative marking in December 2020 in Japan, and thoracic surgeons should investigate the effectiveness of the other numerous techniques for localization of small pulmonary lesions that have been reported.

There are some limitations of the RFID technique in segmentectomy. Sufficient experience and understanding of this system may be required for clinical use. Because the probe has directivity in wireless communication, detection from different directions is required to identify precise RFID marker position. Moreover, careful confirmation of the bronchial diameter before marker placement is advised to avoid intraoperative marker migration. Although we have not experienced dislocation of RFID markers at our institution, removing migrated markers is recommended considering the potential harmful effects associated with residual markers.

In conclusion, for barely palpable pulmonary lesions for which the resection line would be better placed on the adjacent segment to the affected one, RFID markers can provide a complementary method to enable accurate measurement of surgical margins.

## Supplementary Information

Below is the link to the electronic supplementary material.

Supplementary file1 (MP4 246076 KB)
